# Correlation between high-resolution computed tomography features and patients' characteristics in chronic obstructive pulmonary disease

**DOI:** 10.4103/1817-1737.39676

**Published:** 2008

**Authors:** Prem P. Gupta, Rohtash Yadav, Manish Verma, Dipti Agarwal, Manoj Kumar

**Affiliations:** *Department of Respiratory Medicine, Postgraduate Institute of Medical Sciences, Rohtak, India*; 1 *Department of Radiodiagnosis, Postgraduate Institute of Medical Sciences, Rohtak, India*; 2 *Department of Physiology, Postgraduate Institute of Medical Sciences, Rohtak, India*; 3 *Department of Biostatistics, PGIMS, Rohtak, India*

**Keywords:** Chronic obstructive pulmonary disease, correlation analysis, high-resolution computed tomography features

## Abstract

**BACKGROUND:**

During the last few decades, high-resolution computed tomography (HRCT) has come up as a new diagnostic modality to diagnose emphysematous and chronic bronchitis components of chronic obstructive pulmonary disease (COPD). The present study was undertaken to evaluate for various quantitative and qualitative HRCT features in patients with COPD, and to detect patients' characteristics that correlate with these HRCT features.

**MATERIALS AND METHODS:**

Forty male patients with COPD attending the COPD clinic at a tertiary referral hospital and postgraduate medical institute were included in the study. Various HRCT features, including tracheal index, thoracic cage ratio, sterno-aortic distance, thoracic cross-sectional area, vascular attenuation, vascular distortion, mosaic attenuation pattern, and directly visible small airways, were evaluated and correlated with patients' characteristics, including age, duration of illness, quantum of smoking, dyspnea score, quality-of-life index, and various spirometric indices.

**RESULTS:**

We found significant correlations of various quantitative and qualitative HRCT features with age, duration of illness, quantum of smoking, quality-of-life index, and the spirometric indices showing the extent of airways obstruction.

**CONCLUSIONS:**

Various quantitative and qualitative HRCT features were found to correlate with patients' characteristics, spirometric indices, and health-related quality-of-life score, suggesting that HRCT is useful not only in detecting emphysema and its various subtypes but also in predicting the extent and severity of COPD.

Chronic obstructive pulmonary disease (COPD) is a leading cause of morbidity and mortality worldwide. The economic and social burden due to it is both substantial and projected to increase in the coming decades due to continued exposure to COPD risk factors and the changing age profile of the world's population. COPD mortality trends generally track several decades behind smoking trends. In the US in 2000, more women than men died of COPD or its related complications.[1]

COPD consists of a heterogeneous group of disorders, including emphysema, peripheral airways disease, and chronic bronchitis. The diagnosis of COPD is conventionally based upon spirometry;[1] But during the early stage of the disease, conventional spirometry may reveal no abnormality as the earliest changes in COPD affect the alveolar walls and small airways.[2] Also, definition of emphysema is a pathologic one, and it requires histopathological examination to authenticate the disease, which is not usually carried out and not often consented to by the patient.

During the last few decades, with the advent of HRCT as a new diagnostic modality, there has been an increased interest of diagnosing emphysematous and chronic bronchitis components of COPD using HRCT. Few isolated studies have suggested the role of HRCT in diagnosing different entities grouped under COPD. In the present study, apart from evaluating for the HRCT features of COPD, we have tried to detect patients' characteristics, including age, duration of illness, quantum of smoking, dyspnea score, quality-of-life index, and various spirometric indices that are correlated with the various established quantitative and qualitative HRCT features of COPD.

## Materials and Methods

The present study was conducted in the Departments of Respiratory Medicine and Radiodiagnosis at the Postgraduate Institute of Medical Sciences [PGIMS], Rohtak, India. The study was approved by the Institutional Board of Studies and by the Ethical Committee. The patients were enrolled from the COPD Clinic run at the Department of Respiratory Medicine, PGIMS, Rohtak. Eligible male patients with COPD, aged 40 years or more, and who provided explicit written consent, attending the clinic during the period from February 2006 to December 2006 were included. The diagnosis of COPD was based on the criteria defined by ‘*Global Initiative for Chronic Obstructive Lung Disease (GOLD) 2004 update*’.[3] All included patients had a smoking history of more than 20 pack years and had airflow limitation that was not fully reversible (postbronchodilator FEV_1_ less than 80% of the predicted value in combination with FEV_1_/FVC not more than 70%). They had an increase in FEV_1_ less than 200 mL or less than 12% of baseline value 20 minutes after 2 puffs of inhaled salbutamol that was given by a metered dose inhaler through a spacer. The patients were not included in the study if they had any evidence of coexisting bronchiectasis, cystic fibrosis, tuberculosis, bronchial asthma, interstitial lung disease, bronchogenic carcinoma, previous lung surgery, or coronary artery disease. They were excluded if they had any concomitant disorders like diabetes mellitus, chronic alcoholism, uremia, or sarcoidosis.

The study protocol required each patient to undergo routine clinical and laboratory evaluation, including complete blood examination, urine examination, chest radiograph, electrocardiography, echocardiography, and spirometry. Degree of breathlessness was measured by using *Medical Research Council (MRC) dyspnea scale*.[4] Smoking pack years were based on the mode of smoking (*bidi,* cigarette, or *hookka*), daily consumption, and total years smoked. One pack year was 20 cigarettes smoked/day for 1 year.[5] For *bidi,* cigarette equivalents were calculated by multiplying with 0.5 to the quantum of *bidis*;[6] and for hookka, 12.5 g of loose tobacco was equivalent to one packet of 20 cigarettes.[7]

The spirometry was carried out on Transfer Test Model ‘C’, P. K. Morgan, Chatham, Kent, UK. All patients were required to withhold inhaled short-acting bronchodilators 6 hours before test, long-acting β-2 agonists 12 hours before test, and sustained-release theophyline 24 hours before test. Spirometric indices were calculated using the best out of 3 technically satisfactory performances as per recommendations of American Thoracic Society.[8] The following parameters were recorded: PEFR (peak expiratory flow rate) in liters/min, FEV_1_ (forced expiratory volume in the first second) in liters, FVC (forced vital capacity) in liters, FEV_1_/FVC% (forced expiratory volume in first second/forced vital capacity %), SVC (slow vital capacity) in liters, and FEV_1_/SVC% (forced expiratory volume in the first second/slow vital capacity %). A questionnaire, *Airways Questionnaire 30,* validated by previous studies,[9,10] was used to assess the health-related quality of life in the present study [Box 1]. The positive responses were scored and then summed to provide a total score out of 30 (AQ30).

Box 1 **Health-related quality of life questionnaire (AQ 30)**

**Table:** 

		Yes		No		N/A
(1)	Do you suffer from coughing attacks during the day?	□	□	□
(2)	Because of your chest trouble, do you often feel restless?	□	□	□
(3)	Because of your chest trouble, do you feel breathless maintaining the garden?	□	□	□
(4)	Do you worry when going to a friend's house that there might be something there that will set off an attack of chest trouble?	□	□	□
(5)	Do you suffer from chest symptoms as a result of exposure to strong smells, cigarette smoke, or perfume?	□	□	□
(6)	Is your partner bothered by your chest trouble?	□	□	□
(7)	Do you feel breathless while trying to sleep?	□	□	□
(8)	Do you worry about the long-term effects on your health of the drugs that you have to take because of your chest trouble?	□	□	□
(9)	Does getting emotionally upset make your chest trouble worse?	□	□	□
(10)	Because of your chest trouble, are there times when you have difficulty getting around the house?	□	□	□
(11)	Because of your chest trouble, do you suffer from breathlessness carrying out activities at work?	□	□	□
(12)	Do you feel breathless walking upstairs because of your chest trouble?	□	□	□
(13)	Because of your chest trouble, do you suffer from breathlessness doing housework?	□	□	□
(14)	Because of your chest trouble, do you go home sooner than others after a night out?	□	□	□
(15)	Because of your chest trouble, do you suffer from breathlessness when you laugh?	□	□	□
(16)	Because of your chest trouble, do you often feel impatient?	□	□	□
(17)	Because of your chest trouble, do you feel that you cannot enjoy a full life?	□	□	□
(18)	Do you feel drained after a cold because of your chest trouble?	□	□	□
(19)	Do you have a feeling of chest heaviness?	□	□	□
(20)	Do you bother much about your chest trouble?	□	□	□
(21)	Do you have difficulty taking part in sports because of your chest trouble?	□	□	□
(22)	Do you worry about getting an attack of chest trouble even when you are well?	□	□	□
(23)	Are you embarrassed by heavy breathing?	□	□	□
(24)	Does your chest trouble affect you at any time other than when you are having an attack?	□	□	□
(25)	Do you do all the things you want to regardless of the effects on your chest trouble?	□	□	□
(26)	Because of your chest trouble, do you often feel helpless?	□	□	□
(27)	Do you work badly when your chest trouble is bad?	□	□	□
(28)	Because of your chest trouble, do you have difficulty doing housework?	□	□	□
(29)	Is your sex life affected by your chest trouble?	□	□	□
(30)	Do you suffer from discomfort when you cough?	□	□	□

High-resolution computed tomography (HRCT) was carried out using Somatom Plus 4 volume zoom spiral CT scanner, Siemens, Erlanger, Germany. Scanning was performed at a field of view large enough to encompass the patient. Images were obtained at maximum inspiration by using 1 mm collimation at 120 kV (kilo volt) (p) and 90 mA with a 0.75-second acquisition time. Scans were taken at 10-mm intervals with patient in the supine position. Images were reconstructed using a high-spatial frequency algorithm and a 512 × 512 matrix. No contrast was used. Following features were evaluated using HRCT:

1. Tracheal index: A ratio of transverse to anteroposterior diameter at a plane 1 cm above aortic arch. Saber-sheath trachea was described as when the tracheal index was less than 2/3.
2. Thoracic cage ratio: A ratio of anteroposterior to transverse diameter. It was evaluated at two planes: [a] tracheal carina, [b] 5 cm below carina.
3. Sterno-aortic distance: Distance from posterior surface of sternum to anterior margin of aorta at carinal level.
4. Thoracic cross-sectional area: Thoracic cross-sectional area (TCSA) was measured on HRCT images made 1 cm below the top of aortic arch. The ratio of TCSA to the square of height (TCSA/height^2^) was calculated for each patient.
5. Vascular attenuation: Vascular attenuation was considered when there was thinning of pulmonary vessels and reduction in their number.
6. Vascular distortion: Vascular distortion was described as increased branching angles and/or excessive straightening of pulmonary vessels.
7. Mosaic attenuation pattern: Mosaic attenuation meant nonhomogeneous lung density; the latter was described as areas that remain relatively lucent interspersed with areas of normal or higher lung density.
8. Directly visible small airways: The airways with internal diameter not more than 2 mm.

For statistical analyses, group means and standard deviations for each variable were calculated for the entire COPD group. Individual patients having abnormal values compared to normal reference range were identified as having significant HRCT features. Pearsons's coefficient of correlation test and regression analysis were used to analyze the correlations between quantitative HRCT features and the patients' characteristics. Chi-square test was applied to correlate qualitative HRCT features with study variables. Various HRCT features were correlated with PEFR, FEV_1_, FVC, SVC, FEV _1_ /FVC, FEV /SVC, age, duration of illness, smoking pack years, dyspnea grade, and HRQL measured by AQ30 score. All statistical analyses were carried out with the help of SPSS software (version 10.0), Chicago.

## Results

The present study included 40 male patients with COPD. The patients' characteristics were as shown in Table 1. The mean age of the COPD patients was 58.55 ± 7.99 years, and the mean duration of illness was 12.63 ± 6.76 years. The smoking pack years ranged from 20 to 74 years. Their mean dyspnea score was 3.65 ± 0.92. The detailed spirometric values are shown in Table 1. The FEV1 /FVC ratio varied from 29% to 68%, and the oxygen saturation was 88.5% to 98.6%. Thirty-four of the 40 patients had airway questionnaire 30 (AQ30) score between 15 and 25, signifying that these patients were having a quality of life that ranged from poor to average. The patients' characteristics suggest that the patients represented a fairly large spectrum of COPD illness when seen from the severity perspective.

Table 1 also shows the group data of the quantitative HRCT features. The mean tracheal index was 0.67. The mean thoracic cage ratio at carina and 5 cm below the carina was 69.69% and 73.27% respectively. The sterno-aortic distance at carinal level was 3.01 ± 0.86 cm, and the thoracic cross-sectional area 1 cm below the top of aortic arch was 87.57 ± 10.47 cm^2^ /m^2^.

**Table 1:** Group data of COPD patients

Parameter	Minimum	Maximum	Mean	SD
Age, years	40	75	58.55	7.99
Duration-illness, years	2	25	12.63	6.76
Dyspnea scale	2	5	3.65	0.92
Pack years	20	74	33.25	10.71
PEFR, litre/sec	1.5	6.9	3.18	1.38
FEV_1_, litre	0.58	2.90	1.30	0.61
FVC, litre	1.12	4.30	2.53	0.82
FEV_1_/FVC ratio	29	68	50.75	11.22
SVC, litre	1.2	4.4	2.62	0.81
FEV_1_/SVC ratio	29	68	48.54	10.96
O_2_ Saturation, %	88.5	98.6	95.15	2.51
AQ30 score	14	27	20.98	3.41
Tracheal index	0.46	0.94	0.67	0.09
Thoracic cage ratio at carina	61.11	78.10	69.69	4.31
Thoracic cage ratio at 5 cm below carina	62.31	83.08	73.27	4.51
Sterno-aortic distance, cm	1.43	4.55	3.00	0.86
Thoracic cross section Area/ht^2^, cm^2^/m^2^	61.8	104.5	87.57	10.47

**Figure 1:**
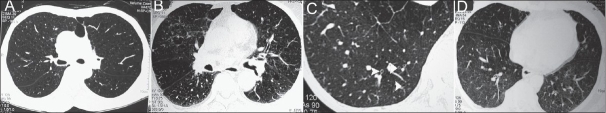
HRCT features in COPD: (A) Vascular attenuation, (B) vascular distortion and increased branching angle, (C) directly visible small airways as ring shadows (white arrowheads), and (D) mosaic attenuation pattern

**Figure 2:**
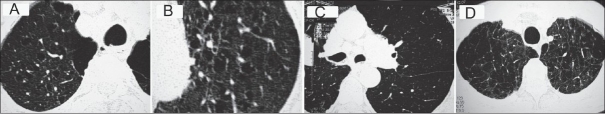
HRCT features in COPD subject showing (A and B) Centriacinar emphysema, (C) panacinar emphysema, and (D) paraseptal emphysema

The HRCT features in individual patients were as shown in Table 2. Saber-sheath trachea described as when tracheal index was less than 0.67 was found in 14 patients. Abnormal thoracic cage ratio was more common at 5 cm below carina (11 patients) than at carina (5 patients). Sterno-aortic distance over 4 cm was found in 5 patients. Large ratio of thoracic cross-sectional area/ht^2^ was present in 28 patients. Among subjective findings, presence of directly visible small airways was the most common finding, followed by vascular attenuation and mosaic attenuation pattern [Figure 1]. Vascular distortion was present only in 8 patients. Vascular distortion and mosaic attenuation pattern were coexisting with vascular attenuation. Among the three types of emphysema, centriacinar emphysema was observed most frequently (16 patients, 40%), followed by distal acinar (13 patients, 32.5%) and panacinar emphysema (11 patients, 27.5%) [Figure 2]. Twenty-five (62.5%) patients had at least one type of emphysema.

**Table 2:** HRCT features in individual patients (*n* = 40)

HRCT features	Number of patients	Percentage of patients
Saber sheath trachea with tracheal index <0.67	14	35.0
Thoracic cage ratio >0.75at Carina	5	12.5
Thoracic cage ratio >0.75 at 5 cm below carina	11	27.5
Sterno-aortic distance >4 cm	5	12.5
Thoracic cross-sectional area/ht^2^ >80.00 cm^2^/m^2^	28	70.0
Vascular attenuation	25	62.5
Vascular distortion	8	20.0
Mosaic attenuation pattern	16	40.0
Directly visible small airways	36	90.0
Centriacinar emphysema	16	40.0
Panacinar emphysema	11	27.5
Paraseptal emphysema	13	32.5
Any type of emphysema	25	62.5

Various HRCT features suggestive of hyperinflation of thoracic cage were evaluated for their possible correlation with patients' characteristics, and the results in detail are displayed in Table 3. Tracheal index was observed to have a highly significant negative correlation with age, duration of illness, pack years, dyspnea scale, and AQ30 score. It had a highly positive correlation with FEV_1_ , FEV_1_/FVC ratio, and FEV_1_/SVC ratio; the correlation with PEFR was also significant. Thoracic cage ratio at carina and the thoracic cage ratio at 5 cm below carina had a highly positive correlation with age, duration of illness, pack years, dyspnea scale, and AQ30 score; they had highly negative correlation with FEV_1_ , FEV_1_/FVC ratio, and FEV_1_/SVC ratio. Similarly, sterno-aortic distance and thoracic cross-sectional area had a highly positive correlation with age, duration of illness, pack years, dyspnea scale, and AQ30 score; they had highly negative correlation with FEV_1_, FEV_1_/FVC ratio, and FEV_1_/SVC ratio.

**Table 3:** Statistical significance [*P* value] of correlation between quantitative HRCT features and study variables (significant correlations are shown in bold)

Parameters	Tracheal index	Thoracic cage ratio at carina	Thoracic cage ratio at 5 cm below carina	Sterno-aortic distance	Thoracic cross sectional area
Age	<0.001	<0.001	<0.001	<0.001	<0.001
Duration of illness	<0.001	<0.001	<0.001	<0.001	<0.001
Dyspnea scale	<0.001	<0.001	<0.001	<0.001	<0.001
Pack years	0.001	<0.001	0.001	<0.001	<0.001
PEFR	0.011	0.011	0.005	0.034	<0.001
FEV_1_	<0.001	<0.001	<0.001	<0.001	<0.001
FVC	0.328	0.098	0.063	0.153	0.006
FEV_1_/FVC ratio	<0.001	<0.001	<0.001	<0.001	<0.001
SVC	0.224	0.078	0.041	0.115	0.005
FEV_1_/SVC ratio	<0.001	<0.001	<0.001	<0.001	<0.001
O_2_ Saturation	0.785	0.825	0.785	0.959	0.427
AQ30 score	<0.001	<0.001	<0.001	<0.001	<0.001

Details of correlation between qualitative HRCT features and the patients' characteristics were as shown in Table 4. The vascular attenuation had a significant correlation with age of the patients, duration of illness, dyspnea scale, smoking pack years, FEV_1_, FEV_1_/FVC ratio, and FEV_1_/SVC ratio. Vascular distortion had no significant correlation with any parameter. The mosaic attenuation pattern correlated significantly with duration of illness, dyspnea scale, smoking pack years, FEV_1_, FEV_1_/FVC ratio, and FEV_1_/SVC ratio. Correlation of presence of directly visible small airways was statistically significant only with dyspnea scale. Centriacinar emphysema had statistically significant correlation with PEFR, FEV_1_, FVC, FEV_1_/FVC ratio, and FEV_1_/SVC ratio. Correlation of presence of any type of emphysema with age, duration, dyspnea scale, pack years, PEFR, FEV_1_, FVC, FEV_1_/FVC ratio, and FEV_1_/SVC ratio was significant.

**Table 4:** Statistical significance [*P* value] of correlation between qualitative HRCT features and study variables (significant correlations are shown in bold)

Parameters	Vascular attenuation	Vascular distortion	Mosaic attenuation pattern	Directly visible small airways	Centriacinar emphysema	Panacinar emphysema	Distal acinar emphysema	Any type of emphysema
Age	0.032	0.396	0.051	0.100	0.182	0.029	0.404	0.032
Duration of illness	0.020	0.098	0.02	0.116	0.061	0.186	0.054	0.020
Dyspnea scale	0.004	0.156	0.003	0.006	0.107	0.173	0.175	0.004
Pack years	0.049	0.206	0.012	0.898	0.347	0.120	0.170	0.049
PEFR	0.050	0.279	0.042	0.957	0.042	0.054	0.081	0.050
FEV_1_	0.044	0.446	0.047	0.801	0.049	0.304	0.279	0.044
FVC	0.045	0.444	0.108	0.918	0.017	0.133	0.120	0.045
FEV_1_/FVC ratio	0.031	0.220	0.048	0.885	0.048	0.060	0.185	0.031
SVC	0.072	0.744	0.269	0.521	0.269	0.321	0.350	0.072
FEV_1_/SVC ratio	0.024	0.055	0.037	0.564	0.047	0.062	0.073	0.024O2
O_2_ Saturation	0.060	0.610	0.072	0.937	0.099	0.108	0.101	0.060
AQ30 score	0.120	0.172	0.197	0.07	0.085	0.501	0.078	0.120

## Discussion

Chronic obstructive pulmonary disease (COPD) is a preventable and treatable disease, with some significant extrapulmonary effects that may contribute to the severity in individual patients. Its pulmonary component is characterized by airflow limitation that is not fully reversible. It is a disease state that has seen significant changes in defining and excluding criteria over the past 50 years[1]; and as a result, some of the earlier studies are not expected to have inclusion criteria of COPD patients as per recent guidelines. There have been lots of variations in the patients' characteristics included in various previous studies. Many studies had selected patients with emphysema only.[11,12] Some studies[13–17] did not have reversibility criteria in accordance with Global Initiative for Chronic Obstructive Lung Disease (GOLD) guidelines. In the present study, all subjects included were smokers and had irreversible or partially reversible airflow limitation, as required for the diagnosis of COPD. The patients having poorly reversible airflow limitation observed in bronchiectasis, cystic fibrosis, tuberculosis, or asthma were not included. This might have led to some variation in the outcome of our study, but the present study has clinical relevance as the diagnosis of COPD was based on the clinical and the spirometric criteria that are invariably used in clinical practice.

We studied the HRCT features which comprised of quantitative and qualitative parameters. Various studies had evaluated quantitative parameters, including tracheal index, sterno-aortic distance, thoracic cage ratio, and thoracic cross-sectional area, that are suggestive of hyperinflated lungs and rib cage seen in COPD. We observed that all these quantitative parameters were correlated significantly with age, duration of illness, dyspnea score, smoking pack years, PEFR, FEV_1_, FEV_1_/FVC ratio, FEV_1_/SVC ratio, and health-related quality-of-life (AQ30) score. No significant correlation was noted with oxygen saturation, which probably was due to the fact that the patients included were having stable course of COPD; 34/40 patients had normal oxygen saturation. Trigaux and co-workers[18] found significant correlation of tracheal index with FEV_1_, FRC, and sterno-spine distance. In a different study, saber-sheath trachea was established as a specific radiographic diagnostic parameter in diagnosis of COPD (specificity, 92.9%), although the sensitivity (39.1%) was low.[14] We found saber-sheath trachea in 14/40 patients (sensitivity 35%). In a study by Arakawa, tracheal index, thoracic cage ratio, and sterno-aortic distance were significantly correlated with FEV_1_/FVC ratio in 74 patients (44 with normal lung function, 30 with COPD).[19] One study[20] showed a good correlation between thoracic cross-sectional area and total lung capacity, functional residual capacity, residual volume, as well as Fletcher's five-degree scale of dyspnea.

In our study, presence of vascular attenuation correlated significantly with duration of illness, smoking pack years, dyspnea score, FEV_1_, FEV_1_/FVC ratio, and FEV_1_/SVC ratio. In a study by Kuwano and co-workers, CT score was assessed by examining low attenuation areas, vascular attenuation, and distortion;[21] it correlated significantly with DL_CO_, FEV_1_/VC ratio, maximum mid-expiratory flow rate, and residual volume/total lung capacity. In another study by MacNee *et al.,* vascular attenuation and distortion on CT scans of 32 patients correlated significantly with DL_CO_, FEV_1_% predicted, and FEV_1_/FVC%.[22]

In the present study, no significant correlation was observed between vascular distortion and various spirometric indices; however, vascular distortion was absent in all 20 patients who had FEV_1_/FVC > 50%. Absence of significant correlation was possibly because we had not carried out grading of vascular distortion. Other studies have found good correlation of grading of vascular distortion with pulmonary function tests.[21,22] It appears from findings of our study and other studies that vascular distortion is present in very advanced cases of emphysema and in those with severely deranged pulmonary functions.

As per study protocol, we studied for mosaic attenuation pattern at end-inspiration. Mosaic attenuation is more pronounced on scans obtained at end-exhalation compared to that found at end-inspiration as in the more conventional end-inspiration technique.[16] This is the probable reason for the detection of mosaic attenuation pattern in only 16 (40%) out of 40 patients in the present study. All these 16 patients had associated features of vascular attenuation and directly visible small airways. Presence of this feature correlated significantly with duration of illness, smoking pack years, dyspnea score, PEFR, FEV_1_, FEV_1_/FVC ratio, and FEV_1_/SVC ratio. Mosaic attenuation pattern in association with other HRCT features is observed to be helpful in distinguishing different disease entities grouped under COPD.[17]

In our study, directly visible small airways were observed as the most prevalent HRCT feature (36/40 patients). Hogg *et al.* showed that despite a decreased radius of small airways, airflow resistance is not significantly increased because of the large number of these peripheral bronchioles.[23] Hence noteworthy disease must be present at the level of peripheral airways, to be detected on conventional pulmonary function tests.[24] Here, HRCT can play an infallible role in a diagnosis of involvement of small airways.

Low attenuation areas due to emphysema were seen by us in 25/40 (62.5%) patients. The presence of this feature correlated significantly with age of patient, duration of illness, smoking pack years, dyspnea score, and spirometric indices. The emphysematous features had upper lobe predominance, explained by the fact that all patients were moderate-to-heavy smokers. In a study by Miniati *et al,,* the HRCT score based on the extent of emphysema correlated with FEV_1_, FVC, forced mid-expiratory flows, maximum flows at 50% and 75% of FVC, residual volume, total lung capacity, and diffusion constant.[25] Klein *et al.*[12] observed that centriacinar emphysema was the dominant parenchymal abnormality and was strongly correlated with DL_CO_ . Similarly, emphysema on CT was observed to be correlated with FEV_1_ and K_CO_ in 20 patients.[26]

We could find only one previous study which explored correlation between HRCT features of emphysema and HRQL.[27] This study used St. George's Respiratory Questionnaire scores and the SF-36 to measure HRQL in patients with alpha_1_-antitrypsin deficiency and found clear relationship between emphysema measured by lung density and HRQL. Our study measured HRQL by using AQ30 in COPD patients and found significant correlation with all measured parameters of lung hyperinflation. In comparison to time-consuming and complicated instruments to measure HRQL, AQ30 included in the present study took significantly less time to complete and score.

To conclude, in the present study, we were able to detect significant correlations between various quantitative HRCT features and patients' characteristics, spirometric indices, as well as health-related quality-of-life score. Moreover, significant correlations were observed using qualitative HRCT features. This suggests that HRCT is helpful not only in detecting emphysema and its various subtypes but also in predicting the extent and severity of the COPD.
